# Multicentric giant cell tumor involving the same foot: A case report and review of literature

**DOI:** 10.4103/0019-5413.32049

**Published:** 2007

**Authors:** Mandeep S Dhillon, AP Prabhudev Prasad, Mandeep S Virk, Sameer Aggarwal

**Affiliations:** Department of Orthopedics, PGIMER, Chandigarh, India

**Keywords:** Foot, giant cell tumor, multicentric

## Abstract

Multicentric giant cell tumour (GCT) is extremely rare; no case has been previously reported where two lesions occurred in the same foot at different sites. We report a case involving the calcaneus and subsequently the 3^rd^ toe of the same foot and review the reported literature. In established cases of multicentricity, the histopathology has to be properly reviewed and the patient has to be followed up for a longer time with serial whole body assessment to pick up any subsquent lesions. The treatment of the local disease does not differ from a standard GCT.

Giant cell tumor (GCT) of bone is a benign aggressive tumor with features of frequent local recurrences and potential for metastasis and malignant transformation.^1^ Nearly 50% of the cases occur in the region of the knee and other frequent sites include the distal radius, proximal humerus and fibula and the pelvic bones.^2^,^3^ Involvement of the small bones of the foot and hand by GCT is rare.^4^,^5^ Unni^4^ has reported an incidence of 1.7% in the hand and 1.2% in the foot. Multicentric giant cell tumor (MCGCT) is even more infrequent, occurring in less than 1% of patients with GCT;^1^,^3^,^6^ more than one lesion may be noted at initial presentation in different anatomic locations or at different times at separate anatomic locations, where local spread cannot be perceived to have occurred. Around 100 cases of MCGCT have been reported in the literature worldwide.^6^–^11^ Though many cases of MCGCT have been reported with involvement of the foot along with different bones in the body, there has been no mention of multicentric nonadjacent site involvement of the same foot. We are reporting one such case.

## CASE REPORT

**Figure 1:**
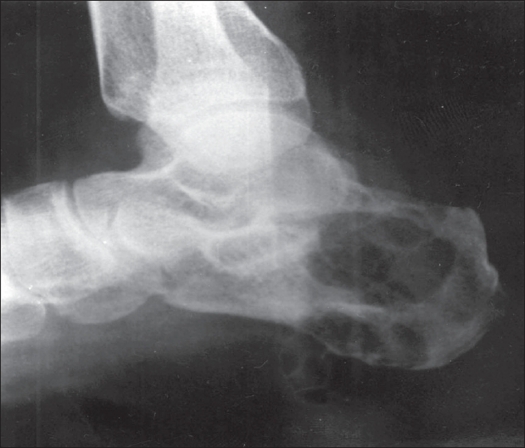
X-ray (lateral view) of the foot showing osteolytic lesion in the calcaneum, with intact articular surfaces

**Figure 2:**
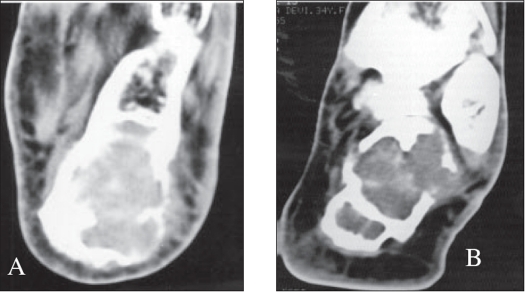
CT scan of the same lesion A) shows expansion of calcaneum. B) Shows cortical breach

**Figure 3:**
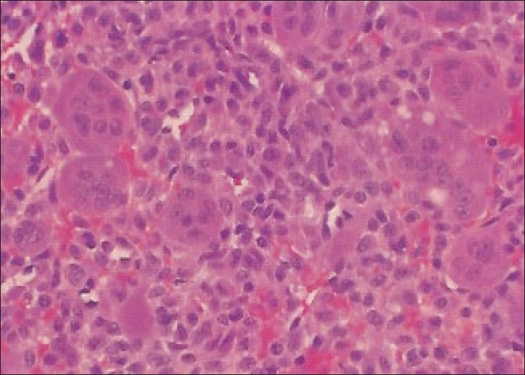
Histopathological picture consistent with giant cell tumor

**Figure 4:**
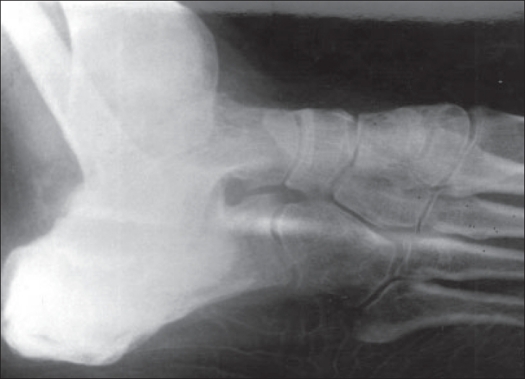
Postoperative X-ray after extended curettage and filling of the cavity with bone cement

A 22-year-old female patient presented in March 2001 with complaints of pain and swelling in the right heel of six months duration. Physical examination revealed a tender, medially prominent bony hard swelling in the hind foot, appearing to arise from the calcaneum. Radiographs showed an expansile osteolytic lesion in the body of the calcaneum with intact articular surface and thinned out surrounding cortex [Figure 1]. A CT scan [Figure 2] of the hind foot showed a 3 cm lesion involving the body of the calcaneum and extending into the tuberosity with cortical breach of the medial and lateral walls. All other bones in the foot were apparently normal. The chest radiograph was normal. Open biopsy showed histological characteristics consistent with GCT [Figure 3]. The calcaneum was approached through a lateral incision; curettage using powered burrs was done and supplemented with chemical cautery using phenol solution. Bone cement was used to fill the cavity and to extend curettage limits chemically and thermally [Figure 4].

**Figure 5:**
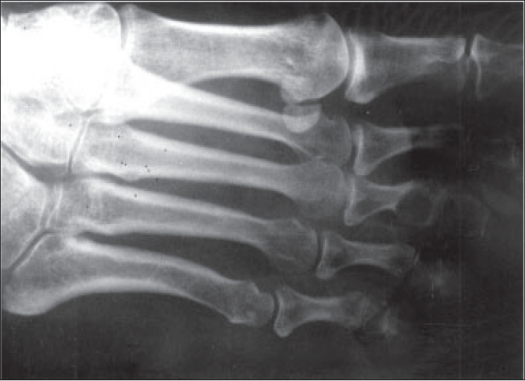
X-ray of the same foot after 18 months showing lytic lesion of the proximal phalanx of third toe

Follow-up was uneventful for 18 months; she then started complaining of pain in the same (right) forefoot at the base of the third toe. Examination revealed swelling and tenderness around the base of the third toe; X-rays showed an osteolytic lesion in the proximal phalanx of the third toe [Figure 5]. Serum biochemistry including serum calcium, phosphate and alkaline phosphatase were done and were within normal limits. Excision biopsy of the local lesion was consistent with GCT with characteristics similar to the previous lesion. A subsequent bone scan was done to identify any other site of involvement in the skeleton which failed to reveal any other lesion. The patient has had a symptom-free follow-up of four years.

## DISCUSSION

GCT of the foot in itself is a rare occurrence.^9^–^11^ Most of these lesions are found in the tarsal bones and involvement of the forefoot bones by GCT is very uncommon. Though involvement of more than one bone in the foot itself is not a rarity we did not find any report wherein the initial lesion started in the foot and remained confined to the foot without a clinically or radiologically identifiable lesion in other parts of the skeleton, as in our case.

MCGCT, when compared to solitary GCT, is encountered more frequently in the short bones of the hand and feet; nevertheless the knee is still the most common site.^3^,^6^,^10^,^12^ Hoch *et al*[13] in their recent report of 30 cases of MCGCT, also found that the incidence of MCGCT is more common in the hand and foot compared to the solitary tumors. Individual lesions in a patient with MCGCT are radiologically and histologically indistinguishable from the solitary GCT.^1^,^6^,^10^,^13^ When there is foot involvement, MCGCT has been known to involve the calcaneum, talus, navicular and cuneiform either as the first or subsequent tumor but involvement of forefoot bones^3^,^6^,^12^,^14^ is very rare [Table 1].

**Table 1:** Literature review of multicentric giant cell tumor with foot involvement

Authors	Number of cases	Age	Male/Female	Number of lesions	Bones involved.
Sim^10^*et al.,* 1977	1	21	F	2	Left lateral cuneiform, L1 vertebral body
Peimer^12^*et al.,* 1980	1	17	F	2	Right tibia, distal phalanx of left hallux
Singson^20^*et al.,* 1983	1	43	M	10	Right and left proximal tibia and flbula, left-sided distal tibia, distal humerus, distal ulna, distal fifth metacarpal, distal fifth phalanx of hand, calcaneus
Hindman^8^*et al.,* 1994	2	22	M	5	Proximal phalanx of left ring finger, left proximal
		11	F	9	fibula, right distal radius, both calcaneum Right metatarsals I-III
Cummins^6^*et al.,* 1996	1	16	F	3	Talus, right distal metaphysis, medial tibial plateu
Dumford^23^*et al.,* 2003	1	16	M	6	Navicular, talus, calcaneum, medial cuneiform, distal tibia and distal femur - all in left side
Park[9] IH *et al.,* 2003	1	19	M	12	Left cuneiform, right proximal femur, both distal femur, both proximal tibia, both distal tibia, left femoral neck, fifth lumbar vertebra, bilateral head and neck of femur

MCGCT tends to involve the younger population compared to solitary GCT, with mean age reported between 20-24 years.^6^,^10^,^13^ The rarity of MCGCT demands a careful scrutiny of other conditions that can present with similar clinico-radiological and histological features. Hyperparathyroidism (Brown tumor) with multiple lytic lesions is a very important differential and it can be differentiated on clinical, radiological and histological grounds.^2^,^10^,^15^ The current case had normal serum calcium, serum phosphate and serum alkaline phosphatase and the histological picture was characteristic of a GCT. Other differential diagnoses worth mentioning are fibrosarcoma, Paget's disease, metastasis, osteosarcoma, multiple myeloma and multifocal infection.[1,2,4,6]

Controversy regarding pathogenesis of MCGCT exists to date. Various mechanisms[6,10,12,15] have been described including contiguous spread, iatrogenic seeding of tumor cells, benign metastasis, malignant transformation and *de novo* multi-focal formation. Synchronous tumors are lesions arising from different locations and are discovered within a short period of time or simultaneously.[6,11,16,17] Metachronous tumors are discovered at different times (usually longer gap) and different places.[18–21] The incidence of the former is more than the latter but the exact duration of time period beyond which to call it metachronous is not defined and a rough arbitrary time period is taken.[6,16,18] Most of the noncontiguous synchronous tumors occurring within the first few years are believed to be benign metastasis to the bone.[6,10,22] Haskell[18] *et al* in their review of literature opined that most of the MCGCTs are synchronous, occurring within a poorly defined time of the initial tumor presentation.

Since MCGCT occurs in less than 1% of GCT, regular screening of GCT patients for multicentricity may not be cost-effective.[6,7] Half-yearly screening by either bone scan or skeletal survey is recommended for GCTs at unusual sites or those diagnosed with multicentric involvement. Our literature review [Table 1] showed that this protocol should be followed for at least five years, as most cases develop multicentricity and additional lesions within this period.

The present case is presented for its rarity, as GCT in the foot is unusual in the first place, while MCGCT isolated to the foot has never been reported. It is hoped that the level of awareness of the average orthopedic surgeon would be increased by this report.

## References

[1] Dahlin DC, Unni KK (1986). Bone tumours. Giant Cell tumour (Osteoclastoma). Springfield, Charles C Thomas.

[2] Szendroi M (2004). Giant cell tumour of bone: A review article. J Bone J Surg Br.

[3] Biscaglia R, Bacchini P, Bertoni F (2000). Giant cell tumours of the bones of the hand and foot. Cancer.

[4] Unni KK (1996). Dahalin's bone tumors: General aspects and data on 11087 cases.

[5] Mirra JM, Picci P, Gold RH (1989). Bone tumors: Clinical radiological and pathological correlations.

[6] Cummins CA, Scarborough MT, Enneking WS (1996). Multicentric giant cell tumor. Clin Orthop.

[7] Taylor KF, Yingsakmongkol W, Conrad KA, Stanton RP (2003). Multicentric giant cell tumor of bone: A case report and review of literature. Clin Orthop.

[8] Hindman BW, Seeger LL, Stanley P (1994). Multicentric giant cell tumor. Report of five new cases. Skeletal Radiol.

[9] Park IH, Jeon IH (2003). Multicentric giant cell tumor of bone: Ten lesions at presentation. Skeletal Radiol.

[10] Sim FH, Dahlin DC, Beabout JW (1977). Multicentric giant cell tumor of bone. J Bone Joint Surg Am.

[11] Madhuri V, Sundaraj GD, Babu NV, Ponnaiya J, Korula RJ (1993). Multicentric giant cell tumor of bone. A report of 2 cases. Indian J Cancer.

[12] Peimer CA, Schiller A, Mankin HJ, Smith RJ (1980). Multicentric giant cell tumor of bone. J Bone Joint Surg Am.

[13] Hoch B, Inwards C, Sundarm M, Rosenberg AE (2006). Multicentric giant cell tumour of bone. Clinicopathological analysis of thirty cases. J Bone Joint Surg Am.

[14] Szendroi M, Antal I, Perlaky G (2000). Mid-foot reconstruction following involvement of five bones by giant cell tumor. Skeletal Radiol.

[15] Taraporvala JC, Goyal DR, Hire D (1997). Multicentric Giant cell tumor of bone- A case report and comprehensive review of literature. Indian J Cancer.

[16] Park Y, Ryu KN, Han C, Bae DK (1999). Multifocal metachronous giant cell tumor of ulna: A case report. J Bone Joint Surg Am.

[17] Tornberg DN, Dick HM, Johnston AD (1975). Multicentric giant cell tumor in the long bones. A case report. J Bone Joint Surg Am.

[18] Haskell A, Wodowoz O, Johnston JO (2003). Metachronous multicentric giant cell tumor: A case report and literature review. Clin Orthop Relat Res.

[19] Wu KK, Mitchel DC, Sprogue HH (1986). Evolution of case of multicentric giant cell tumor over 23 year period. Clin Orthop Relat Res.

[20] Ali MS (1997). Metachronous multicentric giant cell tumor: A case report. Indian J Cancer.

[21] Singson R, Feldman F (1983). Case report 229: Multiple (multicentric) giant cell tumors of bone. Skeletal Radiol.

[22] Campanacci M, Baldini N, Boriani S, Sudanese A (1987). Giant cell tumor of bone. J Bone J Surg Am.

[23] Dumford K, Moore TE, Walker CW, Jaksha J (2003). Multifocal metachronous, giant cell tumor of the lower limb. Skeletal Radiol.

